# Analysis of chromosome 22q11 copy number variations by multiplex ligation-dependent probe amplification for prenatal diagnosis of congenital heart defect

**DOI:** 10.1186/s13039-015-0209-5

**Published:** 2015-12-29

**Authors:** Jingjing Zhang, Dingyuan Ma, Yan Wang, Li Cao, Yun Wu, Fengchang Qiao, An Liu, Li Li, Ying Lin, Gang Liu, Cuiyun Liu, Ping Hu, Zhengfeng Xu

**Affiliations:** State Key Laboratory of Reproductive Medicine, Department of Prenatal Diagnosis, Nanjing Maternity and Child Health Care Hospital Affiliated to Nanjing Medical University, 123# Tianfei Street, Nanjing, 210029 China; Department of Ultrasound, Nanjing Maternity and Child Health Care Hospital Affiliated to Nanjing Medical University, 123# Tianfei Street, Nanjing, 210029 China

**Keywords:** Congenital heart defects, 22q11 deletion syndrome, Prenatal diagnosis, MLPA

## Abstract

**Background:**

Congenital heart defects (CHD) represent one of the most common birth defects. This study aimed to evaluate the value of multiplex ligation-dependent probe amplification (MLPA) as a tool to detect the copy number variations (CNVs) of 22q11 in fetuses with CHD.

**Results:**

A large cohort of 225 fetuses with CHD was screened by fetal echocardiography. Once common chromosome abnormalities in 30 fetuses were screened out by conventional G-banding analysis, the CNVs of chromosome 22q11 in the remaining 195 fetuses were determined by MLPA for prenatal genetic counseling. In 195 CHD fetuses with normal karyotype, 11 cases had pathological CNVs, including 22q11.2 deletion (seven cases), the deletion of 22q11 cat eye syndrome (CES) region (one case), 22q11.2 duplication (one case), 22q13.3 deletion (one case) and 17p13.3 deletion (one case). In total, our findings from MLPA screening represented 4.9 % in our cohort. Among these, three cases were inherited CNVs, and eight cases were *de novo*. These CNVs were further verified by single nucleotide polymorphism (SNP)-array analysis, and their chromosomal location was refined.

**Conclusion:**

This study indicated that MLPA could serve as an effective test for routine prenatal diagnosis of 22q11 in fetuses with CHD.

## Background

Congenital heart defects (CHD) usually refer to the abnormalities in the heart’s structure or function that arise before birth [1]. It represents the most frequent birth defects and the leading cause of death from a congenital structural abnormality worldwide, causing more than 220,000 deaths globally every year [2]. In China, epidemiological studies have suggested a noticeable increase in trend of CHD mortality with the overall mortality rate increasing from 141 in 2003 to 229 in 2010 per 10,000,000 person-years [3].

The pathogenesis of CHD is largely unknown; however, current studies have indicated a multiple interaction between genetic and environmental factors. Specifically, associations between CHD and chromosomal abnormalities have been well recognized, which accounts for about 16 ~ 56 % of CHD [4]. Moreover, copy number variants (CNVs) have also been identified as a significant factor in CHD development and the most common example is the 22q11 deletion syndrome, which is estimated to affect approximately 1/4000 to 1/6000 in live births [5, 6]. In China, fetal echocardiography is performed after the second trimester ultrasound screening. It is well known that abnormal ultrasound finding is one of most common indications for amniocentesis or other invasive examination [7, 8]. Therefore, the amniocentesis or cordocentesis is suggested to diagnose chromosomal abnormalities and CNVs for fetuses with CHD.

Conventional fluorescence in situ hybridization (FISH) with commercial probes (TUPLE1 or N25) has been developed for the prenatal diagnosis of 22q11 chromosome deletion by many prenatal services. Mademont-soler et al. [9] compared FISH and MLPA techniques for detection of 22q11 chromosome deletion. The results showed that the use of MLPA had not increased the number of diagnosis of 22q11 deletion, and the author suggested that MLPA should not replace FISH as a conventional technology for prenatal diagnosis of 22q11 chromosome deletion. However, many reports have pointed out the advantage of MLPA in the postnatal study [10–12]. To date, there is little data about using MLPA in a large cohort prenatal study. In our report, we present a large cohort of 225 CHD fetuses to evaluate the application value of MLPA in prenatal detection of CNVs in 22q11.

## Results

Conventional G-banding analysis detected all 225 fetuses, and then MLPA screening was performed in the remaining fetuses with normal karyotype. In total, chromosomal abnormalities represented 30 cases (13.3 %) in our cohort (Table 1), including 11 fetuses with trisomy 18, 14 fetuses with trisomy 21, one fetus with 45, XO, two fetuses with chromosomal polyploidy, one fetus with 46, XY, add(1) (p36) and one fetus with balanced translocation (46, XY, t(1;2) (p32;q35)) respectively. Of 30 cases with chromosomal abnormalities, 19 cases had isolated CHD and 11 cases had multiple congenital anomalies.

**Table 1:** Summary of aneuploidy and CNVs detected from 225 fetuses with CHD

Types of CHD	Number of fetuses	Number of fetuses with aneuploidy		Number of fetuses with CNVs				Total
			22q11.2 deletion	The deletion of 22q CES region	22q11.2 duplication	22q13.3 deletion	17p13.3 deletion	
Conotruncal defect	85	8	6	1	1	1	1	18
Septal defect	104	17	1	0	0	0	0	18
Left-heart defect	7	0	0	0	0	0	0	0
Right-heart defect	1	0	0	0	0	0	0	0
Other heart defect	28	5	0	0	0	0	0	5
Total	225	30 (13.3 %)	7 (3.1 %)	1 (0.4 %)	1 (0.4 %)	1 (0.4 %)	1 (0.4 %)	41 (17.8 %)

*CHD* congenital heart defects, *CNVs* copy number variants, *CES* cat eye syndrome

**Fig. 1:**
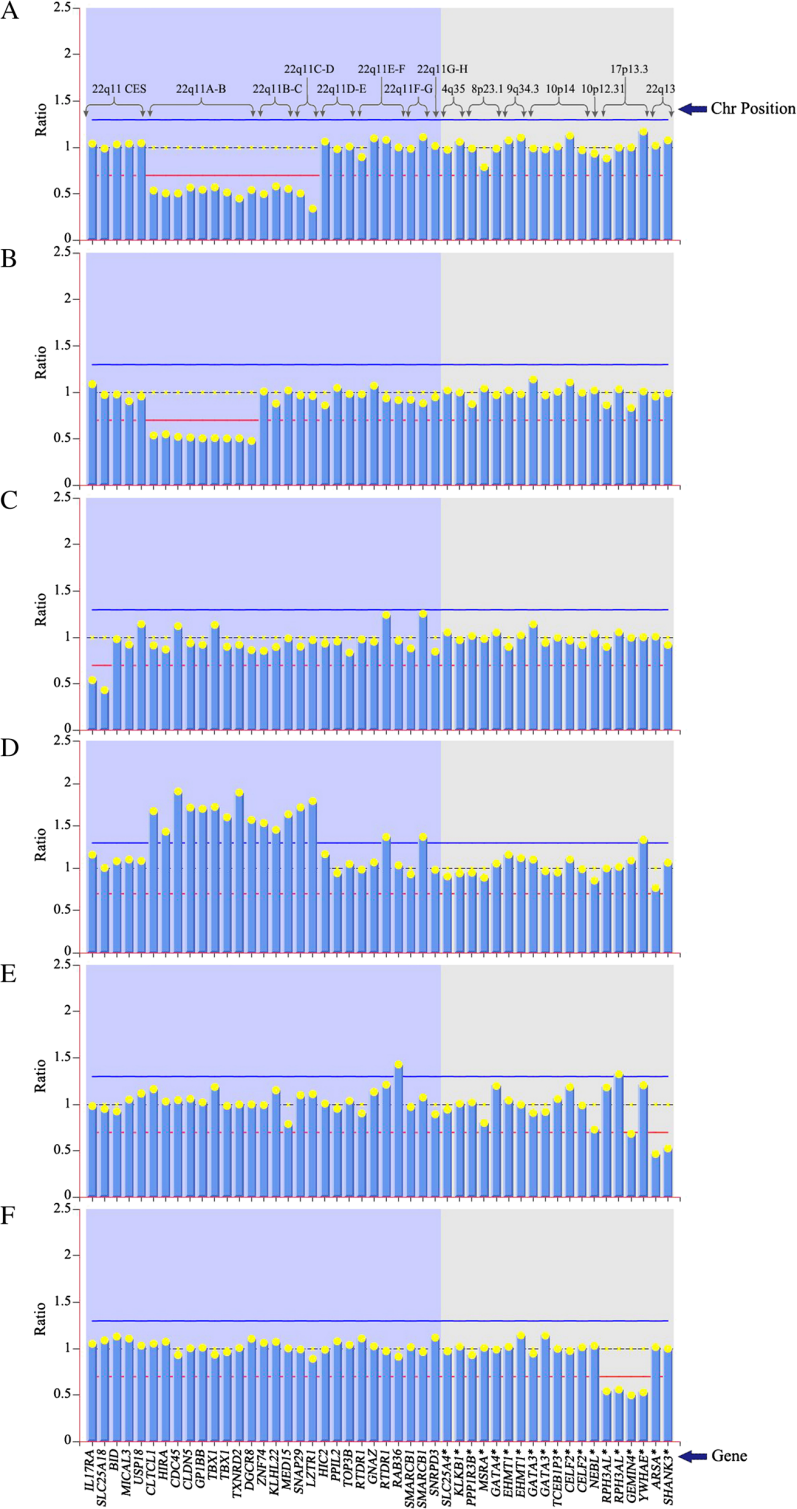
MLPA analysis of chromosome 22q11 in fetuses 1–11. Probe names are shown on the *x*-axis. Their chromosomal location is displayed in the upper panel. Columns corresponding to normalized electropherogram peak areas were calculated using Coffalyser software. **a**. Typical 22q11.2 deletions located from LCR-A to LCR-D regions in fetus 1–6. **b**. 22q11.2 deletions located from LCR-A to LCR-B regions in fetus 7. **c**. The deletion located in the 22q11 cat-eye-syndrome region in fetus 8. **d**. 22q11.2 duplication in fetus 9. **e**. 22q13.3 deletion in fetus 10. **f**. 17p13.3 deletion in fetus 11

**Fig. 2:**
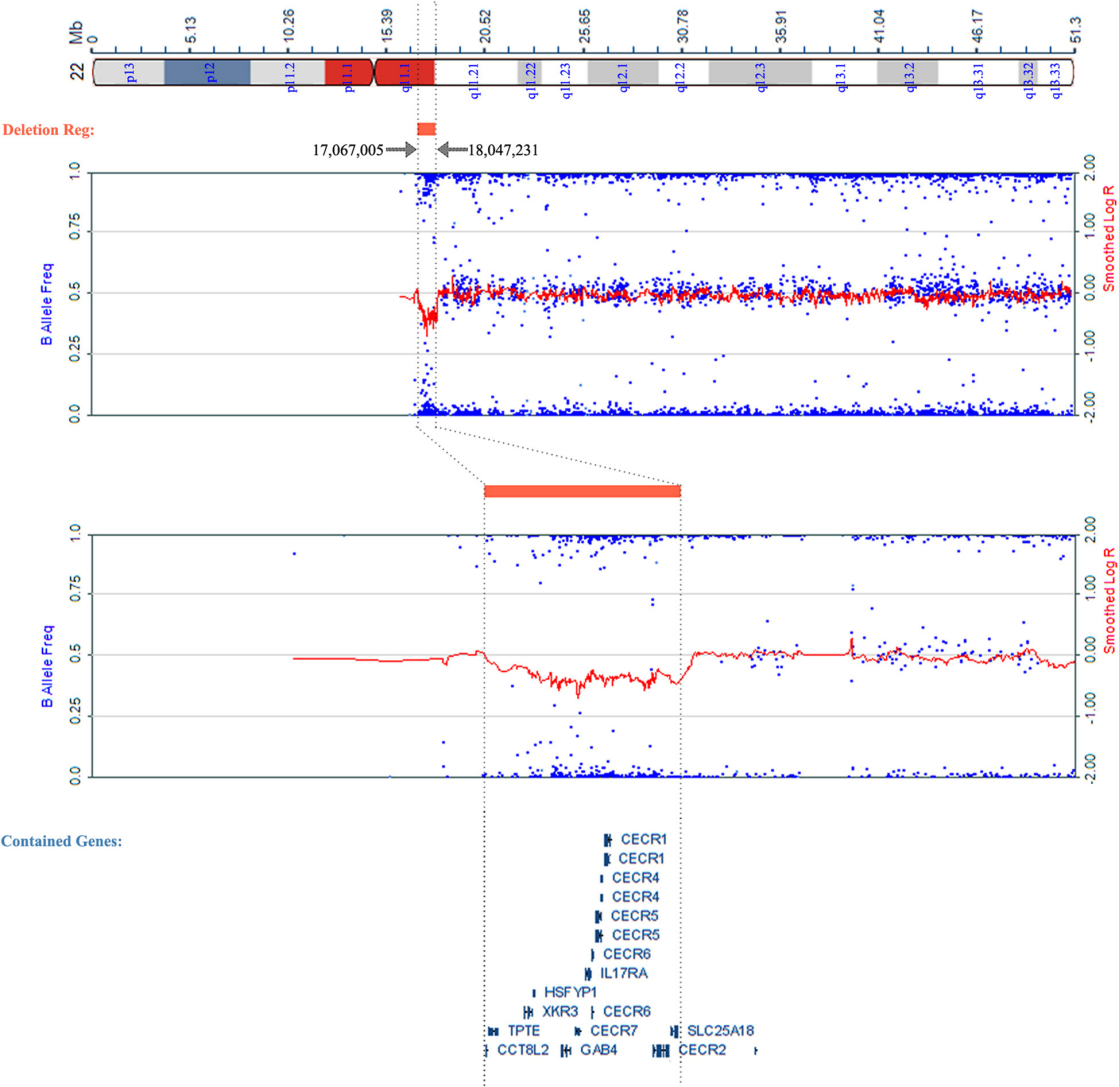
SNP-array analysis of chromosome 22q11 in fetus 8. The analysis revealed a 980 kb heterozygous deletion mapping to position 17,067,005-18,047,231 on chromosome 22q11. Some known genes within the region are indicated in the down panel

For remaining 195 CHD fetuses with normal karyotype, MLPA analysis revealed that 11 cases had CNVs (Table 1). All the 11 cases had isolated CHD, including ten conotruncal defects and one septal defect (Table 1). Fetuses 1–6 showed typical 22q11.2 deletions located from LCR-A to LCR-D regions, fetus seven showed 22q11.2 deletions located from LCR-A to LCR-B regions, and fetus eight showed the deletion based on the 22q11 cat eye syndrome region while Fetus 9–11 showed 22q11.2 duplication, 22q13.3 deletion, and 17p13.3 deletion, respectively (Fig. 1) (Table 2). Succedent SNP-array analysis verified all of the positive results from MLPA and the concordance rate is 100 % (Table 2). For fetus 8, SNP-array analysis revealed a 980 kb heterozygous deletion mapping to position 17,067,005-18,047,231 on chromosome 22q11 (Fig. 2). Further study indicated that each CNV of fetuses 7–9 were inherited from one’s asymptomatic mother or father, while the others were *de novo*.

**Table 2:** MLPA and SNP-array results of 11 fetuses with CHD

Case	Age	Weeks of gestation	Cardiac ultrasound findings	MLPA results			SNP-array results		Type of mutation
				Band	State	Probes	Positon	Size	
1	29	25	IAA,VSD	22q11.2	Del.	CLTCL1 ~ LZTR1	18877787 ~ 21798907	2.92 M	*de novo*
2	24	24	TGA, VSD	22q11.2	Del.	CLTCL1 ~ LZTR1	18877787 ~ 21462353	2.58 M	*de novo*
3	37	25	TA,VSD	22q11.2	Del.	CLTCL1 ~ LZTR1	18877787 ~ 21462353	2.58 M	*de novo*
4	23	27	TA,VSD	22q11.2	Del.	CLTCL1 ~ LZTR1	18895227 ~ 21462353	2.56 M	*de novo*
5	29	24	TOF	22q11.2	Del.	CLTCL1 ~ LZTR1	18877787 ~ 21462353	2.58 M	*de novo*
6	29	23	VSD	22q11.2	Del.	CLTCL1 ~ LZTR1	18895227 ~ 21462353	2.56 M	*de novo*
7	28	23	TOF, PA	22q11.2	Del.	CLTCL1 ~ DGCR8	18895227 ~ 20306993	1.4 M	inherited
8	26	23	DORV, VSD, PA	22q11.122q11.2	Del	IL17RA,SLC25A18	17067005 ~ 18047231	980 k	inherited
9	31	24	AH,VSD	22q11.2	Dup.	CLTCL1 ~ LZTR1	18623108 ~ 21462353	2.8 M	inherited
10	29	24	IAA,VSD, ASD	22q13.3	Del.	ARSA, SHANK3	49045728 ~ 51169045	2.1 M	*de novo*
11	26	22	DORV, VSD, PA	17p13.3	Del.	RH3AL,GEMIN4, YWHAE	18901 ~ 2633324	2.61 M	*de novo*

*VSD* ventricular septal defect, *IAA* interrupted aortic arch, *TGA* transposition of conducting arteries, *TA* truncus arteriosus, *TOF* trilogy of fallot, *PA* pulmonary atresia, *AH* aortic hypoplasia, *ASD* atrial septum defect, *DORV* double outlet right ventricle

## Discussion

In this study, a large cohort of 225 fetuses with CHD was detected by traditional karyotyping and MLPA to identify the chromosome abnormality and CNVs on chromosome 22q11. The results demonstrated 30 fetuses (13.3 %) had a chromosomal abnormality, and 11 fetuses (4.9 %) had CNVs. All positive findings from MLPA were in agreement with those from SNP-array.

Our study used the MLPA P250 DiGeorge kit to identify the CNVs on different chromosomes and found that 3.1 % of CHD cases (7/225) had deletions on 22q11 (Table 1). Several studies have also reported the frequency of 22q11 CNVs in CHD fetuses and the data ranged from 1.6 ~ 11.5 % [4, 13]. Our data was similar to that reported by Moore et al. [14] (17/540, 3.1 %). Of the seven cases with 22q11 deletions, six cases had conotruncal defects. Our results showed high detection rate of 22q11 CNVs in cases with a conotruncal defect (7.1 %, 6/85), which was close to the findings by Galindo et al. [15] (8.7 %) and Bretelle et al. [16] (4.7 %). All these results indicated that the conotruncal defect was mostly associated with 22q11 CNVs [15].

In addition to 22q11, MLPA P250 DiGeorge kit also contains the probes targeting other regions including 22q13, 10p14, 8p23, 9q34, 17p13.3 and 4q34. Thus, we also found the deletions in both 22q13.3 and 17p13.3 (Table 2). The 22q13.3 deletion syndrome was also defined as a Phelan-McDermid syndrome and mainly manifested as global developmental delay, hypotonia, delayed or absent speech, and autistic behavior [17, 18]. Only one study [19] reported one CHD fetus with 22q13.3 deletion by prenatal diagnosis, supporting the findings of our study. The 17p13.3 deletion syndrome is also known as Miller-Dieker Lissencephaly Syndrome. The syndrome is characterized by nervous system anomalies, facial abnormalities, IUGR, mental retardation and other malformation including cardiac defects. To date, at least 29 prenatal cases with the 17p13.3 deletion syndrome have been reported. Among them, only four cases presented with CHD [20]. In our study, the case 11 with 17p13.3 deletion showed cardiac defects including DORV, VSD and PA. These results suggested that MLPA technology could comprehensively and rapidly detect pathogenic CNVs in several different chromosomes regions.

Previous studies found that about 6 ~ 28 % of prenatal 22q11 deletions were inherited from one parent [21]. Two cases with pathological CNVs were inherited from one parent in our study (Table 1). The SNP-array analysis indicated that the position of the fetus was identical with that of the parent. One case in this study had 22q11 duplication inherited from his father (Table 2). Though 22q11 CNVs were present in the three parents, they did not display any mental disorder based on physical examination. Furthermore, their internal organs especially heart revealed no abnormalities by sonographic examination. Several reasons may explain the difference in phenotypes with the same genetic changes, such as allelic variation at the haploid locus, self-repair and environmental effects [22, 23]. Since healthy carriers of chromosomal deletions or duplications have 50 % chance to pass on to the next generation in each pregnancy, two couples who had the fetus with pathogenic CNVs in our study may have a high risk in their next pregnancy. Among the three families, the mother of case seven had a normal child with no CNVs, the mother of case nine had a miscarriage and the mother of case eight had not been pregnant again.

The proximal portion of chromosome 22q was a hot region for chromosomal rearrangement. Cat eye syndrome (CES) is a rare chromosome disorder in human caused by the duplication of chromosome 22q11. The CES critical region covered approximately 2 Mb from the centromere to the locus D22S57 [24], but the deletion of this region is rarely reported. According to DECIPHER database (https://decipher.sanger.ac.uk), only eight cases were detected to carry genomic deletion encompassing the region, and none of them displayed CHD. Kriek et al. [25] firstly reported that a fetus, carrying different rearrangements on chromosome 22q, including the deletion of CES critical region, had the manifestation of developmental delay but no history of cardiac problems. Kriek et al. also suggested that the deletion of CES critical region had little clinical relevance because the normal familial members were carrying this deletion. In our study, we firstly report a fetus with CHD showing a deletion of 980 K spanning the CES region from genomic position 17,067,005 to18,047,231 (Fig. 2). This deletion region contained 14 genes, including six OMIM genes (CECR7, CECR2, CECR1, IL17RA, XKR3, and SLC25A18). Xie et al. [26] identified that IL17RA was related to myocardial disease while there were no other genes having been reported to be related to cardiac disease. Besides the duplication of CES region can cause the manifestation of CHD, our finding firstly indicated a possible relationship between CHD and the deletion of CES region. Further collecting of more cases is still needed to confirm if the deletion of CES region may cause CHD.

## Conclusion

In summary, this study confirmed that MLPA could rapidly and efficiently detect pathogenic CNVs associated with CHD in our cohort. Thus, it is an economical, fast and accurate method for the prenatal genetic diagnosis of CHD for clinical application.

## Methods

### Case recruitment

The study was performed at the Department of Prenatal Diagnosis in Nanjing Maternity and Child Health Care Hospital (Jiangsu, China) between 2011 and 2014. In our hospital, the second trimester ultrasound screening was carried out in all pregnancies. After structural heart defects were found, prenatal echocardiography was offered for detail diagnosis. In total, 225 fetuses presenting with CHD were enrolled in the study. The distribution of different clinical manifestations was shown in Table 1. The CHD cases included in the study were: conotruncal defects (85/225), septal defects (104/225), left-heart defects (7/225), right-heart defect (1/225), and other heart defects (28/225). In all 225 fetuses, 211 cases were with isolated CHD, and the other 14 cases were with extra cardiac anomalies. G-banding analysis was first carried out in all 225 fetuses. Once chromosome abnormalities had been excluded in a fetus with a CHD, the CNV of chromosome 22q11 were investigated by MLPA. The mean age of these pregnancies was 29 ± 4.33 years old and the average gestation age at invasive prenatal diagnosis was 24 ± 2.56 weeks. All the pregnant women signed the informed consent form. This study was approved by the medicine ethics committee of Nanjing Maternity and Child Health Care Hospital.

### Cytogenetic analysis

All samples of amniotic fluid and fetal cord blood were detected using G-banding according to the standard procedure described previously [27].

### Multiplex ligation-dependent probe amplification

Fetal DNA was extracted from uncultured amniotic fluid or fetal blood cells according to the illustrations of the QIAamp DNA Mini Kit (QIAGEN, Hilden, Germany). The SALSA MLPA P250 kit was performed to detect deletion or duplication of the 22q11 chromosomal region. This kit includes 24 probes targeting DiGeorge syndrome region, five probes targeting cat eye syndrome region, and 19 probes for DiGeorge anomaly related chromosomal regions such as 22q13, 10p14, 8p23, 9q34, 17p13.3, and 4q34.

About 100 ~ 150 ng DNA of each sample was involved in the experiment. MLPA analysis was performed following the manufacturer’s instructions. The MLPA products were examined by ABI 3130 genetic analyzer (Thermo Fisher, USA) and quantitative data were analyzed using the software of Coffalyser V8.0 (http://www.mlpa.com/coffalyser). The 30 % increase or decrease of the relative peak area of the probe showed the duplication or deletion of the targeted region, respectively.

### SNP-array analysis

Fetus DNA was examined by Human cyto12 SNP-array scanning (Illumina, USA), which comprised about 300,000 SNPs across the whole genome. SNP-array experiments were carried out as previously described [28] and molecular karyotype analysis was performed by KaryoStudio Software V 1.3.11 (Illumina, USA).

### Consent

Written informed consent was obtained from the pregnant women for publication of this paper. This research was approved by the ethics committee of Nanjing Maternity and Child Health Care Hospital.

## Abbreviations

CHD: congenital heart defects
CNV: copy number variation
MLPA: multiplex ligation-dependent probe amplification
CES: cat eye syndrome
SNP: single nucleotide polymorphism
FISH: fluorescence in situ hybridization
VSD: ventricular septal defect
IAA: interrupted aortic arch
TGA: transposition of conducting arteries
TA: truncus arteriosus
TOF: trilogy of fallot
PA: pulmonary atresia
AH: aortic hypoplasia
ASD: atrial septum defect
DORV: double outlet right ventricle
